# Coevolving parasites and population size shape the evolution of mating behaviour

**DOI:** 10.1186/1471-2148-13-29

**Published:** 2013-02-04

**Authors:** Niels AG Kerstes, Camillo Bérénos, Oliver Y Martin

**Affiliations:** 1 ETH Zürich, Institute of Integrative Biology, Experimental Ecology, Universitätstrasse 16, CH-8092, Zürich, Switzerland; 2 University of Edinburgh, Institute of Evolutionary Biology, Edinburgh, EH9 3JT, UK

**Keywords:** Host-parasite coevolution, Red queen hypothesis, Genetic variation, Multiple mating, Polyandry

## Abstract

**Background:**

Coevolution with parasites and population size are both expected to influence the evolution of mating rates. To gain insights into the interaction between these dual selective factors, we used populations from a coevolution experiment with the red flour beetle, *Tribolium castaneum*, and its microsporidian parasite, *Nosema whitei*. We maintained each experimental population at two different population sizes. We assayed the mating behaviour of both males and females from coevolved and paired non-coevolved control populations after 24 generations of coevolution with parasites.

**Results:**

Males from large, coevolved populations (i.e. ancestors were exposed to parasites) showed a reduced eagerness to mate compared to males from large, non-coevolved populations. But in small populations, coevolution did not lead to decreased male mating rates. Coevolved females from both large and small populations appeared to be more willing to accept mating than non-coevolved females.

**Conclusions:**

This study provides unique, experimental insights into the combined roles of coevolving parasites and population size on the evolution of mating rate. Furthermore, we find that males and females respond differently to the same environmental conditions. Our results show that parasites can be key determinants of the sexual behaviour of their hosts.

## Background

The Red Queen Hypothesis [1,2] proposes that sexual reproduction and non-zero recombination rates in hosts are maintained because of coevolving parasites. As parasites adapt to the most common host genotypes, sex and recombination via crossovers constantly create rare host genotypes that are relatively fit. While it has been shown experimentally that host-parasite coevolution can select for biparental sex in mixed mating populations [3], and that host recombination rates can increase during coevolution [4,5], it is uncertain to what extent multiple matings play a role in maintaining genetic variation in host populations under parasite pressure. In the context of the Red Queen Hypothesis, sexual reproduction is usually directly compared to asexual reproduction, and the possibility of having multiple mates is generally overlooked. It has, however, been shown that polyandry does have the potential to lower offspring parasite load under natural conditions [6].

In tandem with host-parasite coevolution, an intraspecific form of coevolution between the two sexes can also strongly influence mating rates. Sexual conflict relates to the differences between male and female interests over all facets of reproduction [7,8]. Conflicts can, for instance, revolve around the frequency of mating, with males expected to persistently try to increase the number of matings, while females will try to reduce mating rate [9,10]. The influence of coevolution between the sexes is predicted to depend on population size, with larger census size expected to lead to more intense sexual selection and conflict [9]. Larger populations will lead to greater competition between males, as more males will be simultaneously ready to mate. Associated with this, conflict over mating will be more intense, and females will be under greater pressure to resist more frequent superfluous matings. Additionally, greater choice will make remating with the same individuals less likely. The notion that sexual conflict is stronger in larger populations is supported by both theoretical considerations [9] and empirical data [11].

Here, we present results from a host-parasite coevolution experiment with large and small populations of the red flour beetle, *Tribolium castaneum*, that were kept with and without its natural, microsporidian parasite *Nosema whitei*. Previous work on selection lines from our coevolution experiment has provided evidence for a range of phenotypic and genotypic changes in both the host and parasite populations [5,12,13]. *N. whitei* is an obligate killer parasite that infects young *T. castaneum* larvae. In *T. castaneum*, *N. whitei* infection is primarily located in the fat body [14]. Novel infections occur through disintegration of spore-bearing carcasses, or through cannibalism of deceased infected hosts [15,16]. As a consequence, the parasite is not sexually transmitted. Infected individuals of both sexes usually die in the late larval or pupal stages [16], but can survive to adulthood, where they are then found to mate less frequently and to be less fecund [15]. Both sexes of *T. castaneum* mate promiscuously [17], even though females obtain sufficient sperm from a single mating to be fertile for several months, and females do not gain direct benefits from multiple matings [18], at least under standard environmental conditions [19]. After 24 generations of coevolution with parasites, plus two generations of relaxed selection (i.e. parasite-free), we performed mating behaviour assays with coevolved and non-coevolved males and females, from both large and small populations. Focal individuals were paired with a tester individual of the opposite sex, and to characterise mating behaviour we recorded mounting and mating status for an observation period of 25 minutes. A mount does not always result in an actual mating, as the female has to cooperate for successful copulation to occur. We analysed the proportion of pairings that resulted in a mating, the total number of mounts per pairing, and the time until the first mount, as in previous studies in this species [10,20]. We expected to see higher male and lower female [9,11] mating eagerness in large relative to small populations. Both male and female mating eagerness were anticipated to be augmented in coevolved *versus* non-coevolved populations, because an increase in offspring genetic diversity is expected to be beneficial in the context of coevolving parasites [5,21,22].

## Results

### Impact on males

**Figure 1:**
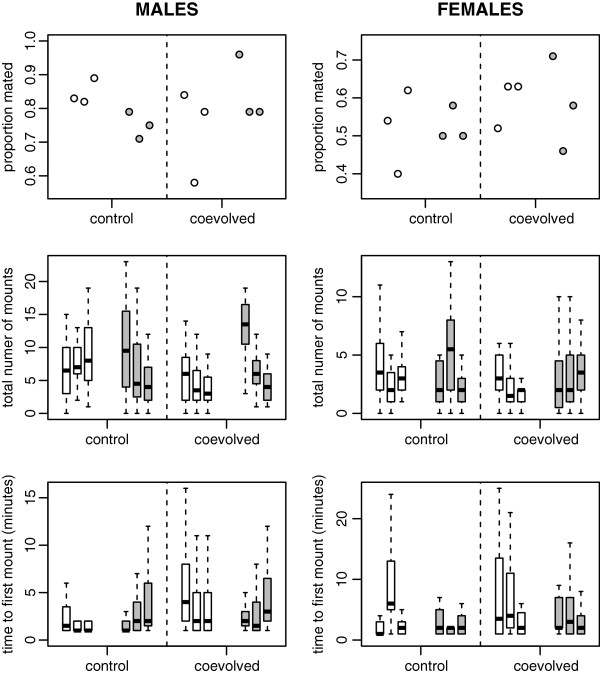
**Results for the three mating behaviour traits assessed: (1) proportion mated, (2) total number of mounts, and (3) time to first mount.** The dots and boxplots show results for each individual experimental line (nr 2, 7 and 8, from left to right). Results for males are shown on the left, results for females on the right. White symbols represent the large populations, grey symbols represent small populations. To improve presentation, boxplot outliers are not shown (they were included in the analyses). In males, interactions between population size and coevolution treatment were present for all traits. Overall, coevolved females appear to be less attractive to tester males. However, this did not result in fewer matings in assays involving coevolved females.

All the results of behavioural assays for beetles of both sexes are shown in Figure 1. For males, we found a significant interaction between population size and coevolution treatment when analysing the proportion of assays that resulted in a mating (Table 1). For the same trait, no significant main effects of treatment (control *versus* coevolution) or population size were found (Table 1). Pairwise contrasts also did not indicate significant differences between control and coevolved populations (large populations: Chi-Square test, Chi-Square = 2.416, P = 0.1201; small populations: Chi-Square = 2.116, P = 0.1458), or between large and small populations (control populations: Chi-Square = 1.818, P = 0.1776; coevolved populations: Chi-Square = 2.823, P = 0.0929).

**Table 1:** GLMMs results for the proportion of successful matings and the total number of times mounting was scored per assay

**Sex**	**Trait**	**Distribution**	**Link function**		**Treatment**	**Size**	**Treatment × size**
males	mated Y/N	Binary	Logit	F(1,258)	0.02	0.01	4.32
				P value	0.8886	0.9113	**0.0386**
	total mounts	Neg Binomial	Log	F(1,258)	7.38	1.85	12.17
				P value	**0.007**	0.1753	**0.0006**
females	mated Y/N	Binary	Logit	F(1,275)	1.03	0.01	0.01
				P value	0.3111	0.915	0.9366
	total mounts	Neg Binomial	Log	F(1,275)	5.3	1.46	2.47
				P value	**0.0221**	0.2281	0.1174

Treatment refers to whether populations coevolved with *N. whitei* or not, and size refers to population size (i.e. small *vs*. large population size). The analysis was performed separately for males and females. Only fixed effects are shown; the random factor 'experimental population' never had a significant effect. Significant P-values are highlighted in bold.

When looking at the total number of mounts, we again found a significant interaction between population size and coevolution treatment (Table 1). No main effect of population size was detected, but the total number of mounts was significantly lower for coevolved males than for control males ('treatment' in Table 1). This effect seems to be mainly caused by the large difference between control and coevolved males from large populations. Indeed, pairwise contrasts indicate that coevolution decreased the total number of mounts in large populations (Mann–Whitney U test, U = 1079, W = 3357, Z = −3.692, P < 0.001), while it had no significant effect in small populations (U = 2266.5, W = 4894.5, Z = −1.304, P = 0.192). Moreover, males from large, coevolved populations mounted significantly less than males from small, coevolved populations (U = 1488.5, W = 3766.5, Z = −3.907, P < 0.001), while there was no significant difference between large and small control populations (U=1632, W = 4260, Z = −1.383, P = 0.167).

For time to first mount we found no significant main effects of coevolution treatment or population size. Similar to the results for the other two traits, for time to first mount there is evidence for an interaction between coevolution treatment and population size, although in this case the interaction did not quite reach significance (Table 2).

**Table 2:** Stratified Cox regression results for the time until the first mount

		**Wald statistic**	**d.f.**	**P**
males	treatment	0.001	1	0.981
	size	0.453	1	0.501
	treatment x size	3.332	1	0.068
females	treatment	0.13	1	0.719
	size	1.61	1	0.205
	treatment × size	0.269	1	0.604

Combining the results across all three traits, coevolution with a parasite thus resulted in a decrease in male eagerness to mate in large populations. In contrast, it had little effect on male eagerness in small populations (Figure 1).

### Impact on females

All behavioural traits measured were simultaneously influenced by both male and female activity. Whereas the total number of mounts and the time to the first mount are assumed to be mainly influenced by males, females need to cooperate for mating to occur and so will influence the actual number of matings [23]. When analysing the results of pairings between our females of interest and tester males, we therefore interpret the total number of mounts and the time to first mount as relative measures of female attractiveness. The proportion of matings is interpreted as a trait that is affected both by tester male eagerness to mate and by the focal female’s willingness to accept a mating. Results show that the total number of mounts was significantly lower in assays with females from coevolved populations than in assays with non-coevolved females (Table 1). Pairwise contrasts indicate a significant difference between coevolved and control large populations (U = 1780, W = 4195, Z = −2.475, P = 0.013), whereas all other comparisons were non-significant (P ≥ 0.198). This finding suggests that females from coevolved populations were less attractive to our tester males than females from paired, non-coevolved populations. At the same time, the proportion of assays that resulted in a mating did not change significantly (Table 1). This result tentatively suggests that coevolved females are more willing to accept mating than their non-coevolved counterparts.

## Discussion

Our results illustrate the importance of both population size and the presence of coevolving parasites for the evolution of mating behaviour. We did not find evidence for the idea that sexual conflict is in general stronger in larger populations [9,11], as male eagerness to mate did not significantly differ between large and small control populations. Interestingly, coevolution with *N. whitei* significantly alters the effect of population size on male mating eagerness. In the case of the males, an interaction between population size and treatment (coevolved *versus* control) was present for all three measured traits. As a result of coevolution with *N. whitei,* the total number of mounts by males decreased in large populations. In contrast, in small populations, coevolution with the parasite did not result in a change in male total number of mounts. Coevolved females appeared to be less attractive to our tester males than control females, as coevolved females were mounted less often than their control counterparts. Yet, the proportion of pairings that resulted in a mating did not differ between control and coevolved females. This observation suggests that coevolved females are more eager to accept a mating once they are mounted by a male.

One possible explanation for the decreased male mating eagerness in large coevolved populations is that female fitness under parasite conditions is more variable than under parasite-free conditions. *N. whitei* is known to cause strongly reduced fecundity in *T. castaneum*[14,15]. In fact, moderately to severely infected mated females were found not to produce any eggs [24]. Infected females should therefore be less preferred mates than non-infected females. Hence, males could benefit from being able to determine infection status of females. The observed lower male eagerness to mate could thus be explained by males being more selective towards females. In small populations there might have been insufficient variation in female fitness for males to evolve increased selectivity. Alternatively, host-parasite coevolution could have led to local adaptation [25]. If that were the case, the observed reduced mating eagerness might in fact reflect a preference for sympatric females. No clear patterns of local adaptation were found in different lines from our coevolution experiment [13]. Higher resistance evolved in coevolved lines in a directional and unspecific way [13]. Yet, testing our males against sympatric and allopatric females would be necessary to clarify the relevance of this hypothesis.

In small populations, coevolution did not lead to decreased male mating eagerness. Due to drift and selection, variation in fitness in these populations might have been too small to select for choosier males. Indeed, our small populations were found to harbour less genetic diversity than paired large populations [12]. In addition, multiple mating can act as a mechanism to counter inbreeding effects in this species [23]. Negative inbreeding effects [26,27] could have prevented a decline in mating eagerness in small, coevolving populations. Inbreeding by itself does not have negative effects on male mating success in *T. castaneum*[28].

The observation that coevolved females seemed more willing to accept a mating than non-coevolved females suggests that females benefited from creating more genetic diversity in their offspring during host-parasite coevolution. As population size did not have an effect on female mating behaviour, it is possible that negative frequency-dependent selection by the parasites [29-31] contributed to this result. Variable selection, caused by temporal fluctuations in the environment, is hypothesized to favour polyandry [32]. In *T. castaneum*, changing the environmental context can in fact alter the costs and benefits of polyandry [19]. Multiple mating increases genetic diversity in the offspring, and thus the chance that at least some of them will survive and reproduce in the next generation. Negative frequency-dependent selection by parasites can create such an ever-changing environment [33,34], and could therefore select for more promiscuous females. This idea would match the observation that recombination rates in our experimental populations were found to have increased during coevolution, presumably as a result of fluctuating selection exerted by the parasites [5].

Dissimilar evolutionary responses of the sexes can be attributed to sexual conflict, or to a possible disparate effect of infection on the two sexes. In fact, males and females of the same species are often found to differ in their ability to cope with infection [35]. Infected males that survived to adulthood might for example have suffered less from reduced reproductive success than infected females. This could explain why coevolved males in large populations became choosier, while coevolved females did not. However, males are in general more susceptible to parasites than females [36]. In *T. castaneum*, prevalence of tapeworm (*Hymenolepis diminuta*) infection was indeed observed to be higher in males than in females [37], and tapeworm infection had more severe fitness effects on males than on females [38]. Infected males were found to have reduced mating vigor, as well as decreased sperm production and sperm competitive ability [39].

In *T. castaneum*, resistance to infection with tapeworm was found to be associated with fitness costs [40]. In closely related mealworm beetles (*Tenebrio molitor*) mating was found to induce the down-regulation of immune function [41]. In addition, yellow dung flies (*Scathophaga stercoraria*) evolved larger reproductive organs and reduced immune (phenoloxidase) responses under polyandrous *versus* monogamous conditions [42]. If such a negative association between mating success and immune function also exists in *T. castaneum*, then one would expect to see reduced mating rates in beetles from coevolved populations. It was found that coevolved lines from our long-term coevolution experiment evolved higher resistance against parasite infection [43]. Males - the more susceptible sex - might suffer relatively more from parasitism under more promiscuous mating conditions than females. A sex-specific difference in the trade-off between mating success and immunity might be an alternative explanation for the dissimilar response of the two sexes to coevolution with *N. whitei*, as observed in large populations. Interestingly, tapeworm infection only reduced male fitness and female fecundity under high intraspecific competition, not under low intraspecific competition [38]. The expected difference in intraspecific competition between our large and small populations could thus have influenced the effect of infection on fitness, the trade-off between mating success and immunity, and consequently the evolution of mating behaviour.

In addition, a specific trade-off between the investment in immunity and investment in mating might explain our female results. Possibly, coevolved females, which have increased resistance against *N. whitei* infection, produce less sex pheromones than their control counterparts, thus reducing their attractivity to our tester males. At the same time, they might invest less in mate choice and mate rejection, thus making them appear more eager to accept a mating once mounted.

There are many examples of parasites that manipulate the behaviour of their hosts to increase transmission success (see [44]). For instance, mammals infected with rabies virus are more likely to approach and bite other animals, thus infecting new hosts [45]. Ants infected with the liver fluke (*Dicrocoelium lanceolatum*) climb to grass tops, where they are exposed to a greater risk of being eaten by the parasite’s final host [46]. Also the host’s mating behaviour might be manipulated by parasites. For instance, *Drosophila* males that are infected with *Wolbachia* mate at a higher rate than uninfected males [47]. In addition, there is some evidence for the idea that sexually transmitted parasites increase their insect host’s mating rate [48]. Most of the known parasite-related changes in host behaviour are direct, i.e. the parasite manipulates the behaviour of its current host. Host behaviour can, however, also be affected by coevolving parasites in a non-manipulative manner, such that behavioural changes represent an adaptive response to parasite pressure. Mate choice in humans is for example influenced by MHC (major histocompatibility complex) genes [49], which play an important role in immune recognition. Moreover, a number of mathematical studies suggest that sexually transmitted parasites can select for reduced host promiscuity under certain conditions [48]. By testing the effect of coevolution with a parasite in naïve hosts (i.e. hosts that were kept parasite-free for two generations), this study presents unique, experimental insights into the non-manipulative role of coevolving, non-sexually transmitted parasites on host mating behaviour. Our data provide evidence for the interpretation that both host-parasite coevolution and population size shape the evolution of mating behaviour. When investigating mating behaviour in natural populations, the impact of both factors should therefore be considered. The possibility for hosts to counteract adapting parasites via increased mating rates might be limited by a trade-off between mating success and immunity. Increasing recombination rates might therefore be a less costly alternative for increasing offspring genetic diversity.

## Conclusions

This study provides unique, experimental insights into the combined roles of coevolving parasites and population size on the evolution of mating behaviour. Overall, our findings illustrate that parasites can interact with population characteristics to determine the evolution of sexual behaviour of their hosts. This is particularly noteworthy as coevolution with parasites led to altered mating behaviour even though the parasite in question is not sexually transmitted. Furthermore, we find that males and females respond differently to the same environmental conditions.

## Methods

Eight *T. castaneum* populations, which each originated as a hybrid cross between two stock lines [43], were split up and assigned to two treatments: a parasite-free control, and a coevolution treatment with *N. whitei*. Each original population was thus present in both treatments. In addition, each population, in each treatment, was maintained at two different population sizes. In the small populations, every generation 50 unsexed beetles were randomly picked to generate the next generation. In the large populations, 500 unsexed beetles were picked to found the new generation. More details about the long-term coevolution experiment are described in [43]. Beetles from the 24^th^ generation of the coevolution experiment were used for tests. Infection with *N. whitei* has been found to decrease mating frequency in *T. castaneum*[15]. All beetle populations, including those from the coevolution treatment, were therefore kept under parasite-free conditions for two additional generations. Adults from both coevolved and control populations were allowed to deposit eggs in parasite-free medium. After 5 days we removed the adults from the medium, thus making sure that the medium would remain parasite-free. Because no dead individuals were left to disintegrate or to be cannibalized on, we could prevent spores from possibly infected individuals to be transmitted to the next generation, Additionally, offspring were monitored for signs of infection with *N. whitei* (e.g. the presence of dead larvae). Next, we selected three populations (corresponding to nrs. 2, 7 and 8 in [43]), and collected male and female pupae from both coevolution treatments and population sizes. Six weeks after collecting the pupae, the emerged adult beetles were used in mating assays. Individual beetles were paired with one Georgia1 (see [10,19,20]) tester beetle of the opposite sex to assess male and female effects separately against a standard background. Every minute, for a total of 25 minutes, we scored mounting of the female by the male. In case a male mounted a female for more than 30 consecutive seconds, we scored a successful mating. Per population, per treatment, per population size, and per sex, we performed up to 24 unique mating assays, resulting in a total of 545 assays. In some lines we were not able to collect enough individuals, or individuals died after collection.

All analyses were performed for males and females separately, as our experiments were designed to assess specific male and female effects in isolation. We used generalized linear mixed models (GLMM) to analyse differences in (a) the proportion of the assays that resulted in a mating (distribution: binary, link: logit), and (b) the total number of times that mounting was positively scored (distribution: negative binomial, link: log). Both models included coevolution treatment, population size, and the interaction between the two as fixed factors, and population as a random factor. GLMMs were performed using the GLIMMIX procedure in SAS (SAS Institute Inc.). In addition, we analysed pairwise comparisons for the effects of population size and coevolution treatment on total mounts via Mann–Whitney U tests (IBM SPSS Statistics, version 19), and on the proportion of assays that resulted in a mating via Chi-square tests (IBM SPSS Statistics, version 19). We used stratified Cox regressions to analyse the time to the first mount (IBM SPSS Statistics, version 19). Coevolution treatment, population size and the interaction between the two were included as covariates. The model was stratified according to population.

## Competing interests

The authors declare that they have no competing interests.

## Authors’ contributions

CB performed the coevolution experiment, NAGK and OYM performed the mating assays, analysed the data, and wrote the paper. All authors read and approved the final manuscript.
